# Stress and pain regulation: Parallel processes among traumatized individuals

**DOI:** 10.1192/j.eurpsy.2023.432

**Published:** 2023-07-19

**Authors:** K. Ginzburg, R. Defrin

**Affiliations:** Tel Aviv University, Tel Aviv, Israel

## Abstract

**Introduction:**

The human reaction to traumatic events is often marked by a dialectic alteration of two emotional states –a state characterized by intrusion, anxiety and hyperarousal, and a state of denial marked by dissociation and numbing. These two seemingly opposite states represent attempts to modulate stress, as gradual reduction of their intensity represents an adaptation to stress. Maladaptive reactions to trauma, however, reflect disrupted regulation capacities, manifested as persistent over-modulation or under-modulation of stress.

**Objectives:**

To demonstrate that these manifestations of disrupted regulation, as observed among individuals with posttraumatic stress disorder (PTSD) and borderline personality disorder (BPD) are also reflected in patterns of pain modulation.

**Methods:**

Three studies using self-report questionnaires and psychophysical tests, assessing sensitivity to pain, as reflected by pain thresholds, and reactivity to suprathreshold noxious stimuli, as implicated in their rating

**Results:**

Study 1 Included 32 PTSD outpatients, 29 anxiety disorder outpatients, and 20 healthy controls. PTSD patients reported higher rates of chronic pain (83.3%) than anxiety patients (42.0%) and controls (5.0%). PTSD severity correlated with chronic pain severity (*r* = 0.61, *p* < 0.01). PTSD patients displayed a unique paradoxical pain profile, according to which their pain thresholds were significantly *higher* than those of the anxiety patients and controls (p < 0.01), but they perceived suprathreshold stimuli as being much *more intense* (*p* < 0.01).

**Study 2** included 32 PTSD outpatients and 43 healthy controls. Findings replicated the paradoxical pain profile among PTSD patients. Pain thresholds were positively associated with dissociation level (*b* = 0.49; *p* < 0.05) and negatively associated with anxiety level (*b* = -0.63, *p* < 0.01). Pain ratings were positively associated with anxiety (*b* = 0.52, *p* < 0.05) and negatively related to dissociation levels (*b* = -.51, *p* < 0.05).

**Study 3** included 46 women diagnosed with BPD and 47 healthy controls. Women with BPD reported higher levels of childhood trauma (*p* < 0.05) than the controls. They also demonstrated higher pain thresholds (*p* < 0.05). Among subjects with high levels of body dissociation, implicated by reduced body awareness, those with BPD demonstrated *hyposensitivity* to pain, manifested in higher pain thresholds, lower suprathreshold pain ratings, and pain evoked by higher temperature, than the controls. Among those with low levels of body dissociation, BPD subjects demonstrated *increased reactivity* to pain as manifested in higher pain ratings and pain evoked by lower temperature.

**Conclusions:**

These findings demonstrate the association between over-modulation and under-modulation of stress and over-modulation and under-modulation of pain, respectively, among PTSD and BPD patients. These findings point to parallel processes of disrupted regulation among traumatized individuals.

**Disclosure of Interest:**

None Declared

